# *N*′-(2-Chloro­benzyl­idene)-4-methyl­benzohydrazide

**DOI:** 10.1107/S1600536812024099

**Published:** 2012-05-31

**Authors:** De-Cheng Wang, Xiao-Rong Li, Lei Gao, Yong Hai, Jiang-Ning Wang

**Affiliations:** a Capital Medical University, Beijing 100069, People’s Republic of China; b Central Laboratory, Luhe Teaching Hospital of the Capital Medical University, Beijing 101100, People’s Republic of China; c Beijing Chao-Yang Hospital, Beijing 100020, People’s Republic of China

## Abstract

In the title compound, C_15_H_13_ClN_2_O, the mol­ecule displays a *trans* conformation with respect to the C=N bond. The two aromatic rings form a dihedral angle of 12.0 (3)°. In the crystal, mol­ecules are connected *via* N—H⋯O hydrogen bonds into chains propagating along the *c*-axis direction.

## Related literature

For the crystal structures of hydrazones, see: Wardell *et al.* (2006 ▶); Kummerle *et al.* (2009 ▶). For bond-length data, see: Allen *et al.* (1987) ▶.
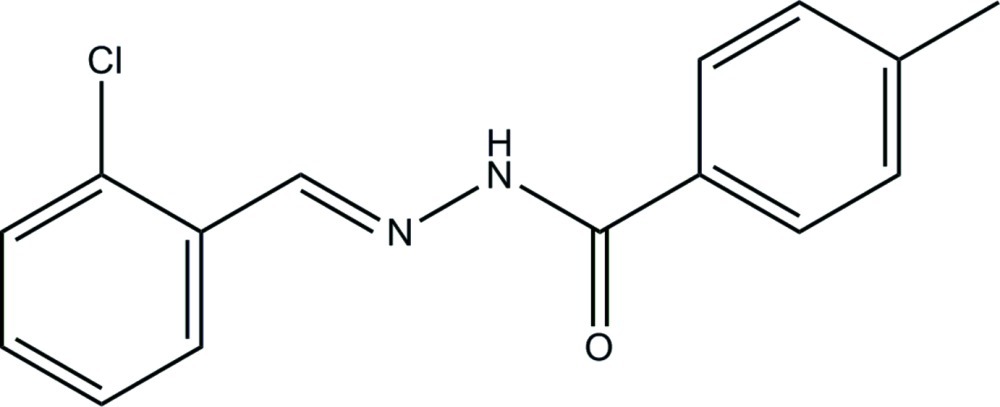


## Experimental

### 

#### Crystal data

C_15_H_13_ClN_2_O
*M* *_r_* = 272.72
Monoclinic, 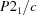

*a* = 11.0697 (14) Å
*b* = 13.4436 (16) Å
*c* = 9.1643 (11) Å
β = 96.576 (2)°
*V* = 1354.8 (3) Å^3^
*Z* = 4
Mo *K*α radiation
μ = 0.28 mm^−1^
*T* = 298 K
0.10 × 0.10 × 0.07 mm

#### Data collection

Bruker SMART CCD diffractometer
Absorption correction: multi-scan (*SADABS*; Sheldrick, 1996) ▶ *T*_min_ = 0.973, *T*_max_ = 0.981
11486 measured reflections
2096 independent reflections
1682 reflections with *I* > 2σ(*I*)
*R*_int_ = 0.026
θ_max_ = 23.9°

#### Refinement

*R*[*F* ^2^ > 2σ(*F* ^2^)] = 0.037
*wR*(*F* ^2^) = 0.104
*S* = 1.03
2096 reflections
176 parameters
1 restraint
H atoms treated by a mixture of independent and constrained refinement
Δρ_max_ = 0.16 e Å^−3^
Δρ_min_ = −0.18 e Å^−3^

### 

Data collection: *SMART* (Bruker, 1998 ▶); cell refinement: *SAINT* (Bruker, 1998 ▶); data reduction: *SAINT*; program(s) used to solve structure: *SHELXS97* (Sheldrick, 2008 ▶); program(s) used to refine structure: *SHELXL97* (Sheldrick, 2008 ▶); molecular graphics: *SHELXTL* (Sheldrick, 2008 ▶); software used to prepare material for publication: *SHELXTL*.

## Supplementary Material

Crystal structure: contains datablock(s) global, I. DOI: 10.1107/S1600536812024099/hb6816sup1.cif

Structure factors: contains datablock(s) I. DOI: 10.1107/S1600536812024099/hb6816Isup2.hkl

Supplementary material file. DOI: 10.1107/S1600536812024099/hb6816Isup3.cml

Additional supplementary materials:  crystallographic information; 3D view; checkCIF report

## Figures and Tables

**Table 1:** Hydrogen-bond geometry (Å, °)

*D*—H⋯*A*	*D*—H	H⋯*A*	*D*⋯*A*	*D*—H⋯*A*
N2—H2⋯O1^i^	0.90 (1)	2.05 (1)	2.8976 (19)	159 (2)

Symmetry code: (i) 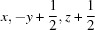.

**Fig. 1.**
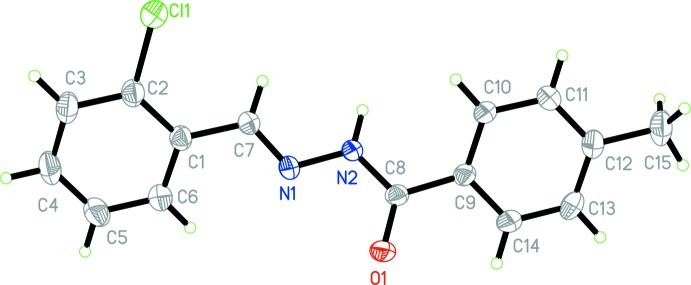
The molecular structure of the title compound showing displacement ellipsoids drawn at the 30% probability level.

**Fig. 2.**
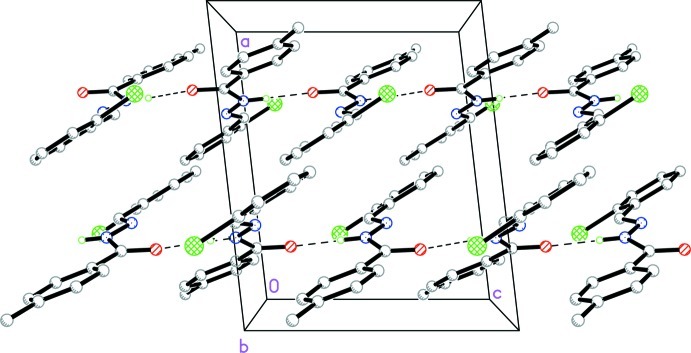
The crystal packing of the title compound viewed along the b axis. Dashed lines show intermolecular hydrogen bonds.

## supplementary crystallographic information

### Comment

Recently, a number of hydrazones have been prepared and structurally characterized (Wardell *et al.*, 2006; Kummerle *et al.*, 2009). As an extension of work on the structural characterization of hydrazones, the title compound, Fig. 1, is reported here.

The molecule of the compound displays a *trans* conformation with respect to the C=N bond. The two aromatic rings form a dihedral angle of 12.0 (3)°. The bond lengths are within normal ranges (Allen *et al.*, 1987). In the crystal, molecules are connected *via* intermolecular N—H···O hydrogen bonding (Table 1) into chains along the *c* axis (Fig. 2).

### Experimental

2-Chlorobenzaldehyde (0.1 mmol, 14.0 mg) and 4-methylbenzhydrazide (0.1 mmol, 15.0 mg) were stirred in 20 ml methanol at room temperature for 30 min. A large number of colorless blocks were formed by slow evaporation of the methanolic solution containing the compound in air.

### Refinement

The amino H atom was located from a difference Fourier map and refined isotropically, with N–H distance restrained to 0.90 (1) Å. The remaining hydrogen atoms were positioned geometrically and treated as riding on their parent atoms, with C–H distances of 0.93–0.96 Å, and with *U*_iso_(H) = 1.2U_eq_(C_aromatic_) and 1.5U_eq_(C_methyl_).

### Crystal data

**Table:**

C_15_H_13_ClN_2_O	*F*(000) = 568
*M**_r_* = 272.72	*D*_x_ = 1.337 Mg m^−^^3^
Monoclinic, *P*2_1_/*c*	Mo *K*α radiation, λ = 0.71073 Å
Hall symbol: -P 2ybc	Cell parameters from 5323 reflections
*a* = 11.0697 (14) Å	θ = 2.4–24.3°
*b* = 13.4436 (16) Å	µ = 0.28 mm^−^^1^
*c* = 9.1643 (11) Å	*T* = 298 K
β = 96.576 (2)°	Block, colorless
*V* = 1354.8 (3) Å^3^	0.10 × 0.10 × 0.07 mm
*Z* = 4	

### Data collection

**Table:**

Bruker SMART CCD diffractometer	2096 independent reflections
Radiation source: fine-focus sealed tube	1682 reflections with *I* > 2σ(*I*)
Graphite monochromator	*R*_int_ = 0.026
ω scans	θ_max_ = 23.9°, θ_min_ = 2.4°
Absorption correction: multi-scan (*SADABS*; Sheldrick, 1996)	*h* = −11→12
*T*_min_ = 0.973, *T*_max_ = 0.981	*k* = −15→15
11486 measured reflections	*l* = −9→10

### Refinement

**Table:**

Refinement on *F*^2^	Primary atom site location: structure-invariant direct methods
Least-squares matrix: full	Secondary atom site location: difference Fourier map
*R*[*F*^2^ > 2σ(*F*^2^)] = 0.037	Hydrogen site location: inferred from neighbouring sites
*wR*(*F*^2^) = 0.104	H atoms treated by a mixture of independent and constrained refinement
*S* = 1.03	*w* = 1/[σ^2^(*F*_o_^2^) + (0.051*P*)^2^ + 0.4392*P*] where *P* = (*F*_o_^2^ + 2*F*_c_^2^)/3
2096 reflections	(Δ/σ)_max_ < 0.001
176 parameters	Δρ_max_ = 0.16 e Å^−^^3^
1 restraint	Δρ_min_ = −0.18 e Å^−^^3^

### Special details

**Table:**

Geometry. All esds (except the esd in the dihedral angle between two l.s. planes) are estimated using the full covariance matrix. The cell esds are taken into account individually in the estimation of esds in distances, angles and torsion angles; correlations between esds in cell parameters are only used when they are defined by crystal symmetry. An approximate (isotropic) treatment of cell esds is used for estimating esds involving l.s. planes.
Refinement. Refinement of F^2^ against ALL reflections. The weighted R-factor wR and goodness of fit S are based on F^2^, conventional R-factors R are based on F, with F set to zero for negative F^2^. The threshold expression of F^2^ > 2sigma(F^2^) is used only for calculating R-factors(gt) etc. and is not relevant to the choice of reflections for refinement. R-factors based on F^2^ are statistically about twice as large as those based on F, and R- factors based on ALL data will be even larger.

### Fractional atomic coordinates and isotropic or equivalent isotropic displacement parameters (Å^2^)

**Table:**

	*x*	*y*	*z*	*U*_iso_*/*U*_eq_	
Cl1	0.73671 (7)	−0.12300 (4)	0.12887 (7)	0.0804 (3)	
N1	0.68758 (14)	0.16431 (11)	−0.06660 (16)	0.0437 (4)	
N2	0.73084 (15)	0.24311 (11)	0.02057 (16)	0.0440 (4)	
O1	0.76428 (13)	0.33102 (10)	−0.18063 (13)	0.0560 (4)	
C1	0.62975 (16)	−0.00454 (13)	−0.0883 (2)	0.0444 (5)	
C2	0.65016 (18)	−0.10128 (14)	−0.0383 (2)	0.0508 (5)	
C3	0.6060 (2)	−0.18270 (15)	−0.1196 (2)	0.0611 (6)	
H3	0.6209	−0.2467	−0.0835	0.073*	
C4	0.5403 (2)	−0.16849 (17)	−0.2538 (3)	0.0673 (6)	
H4	0.5098	−0.2229	−0.3088	0.081*	
C5	0.5195 (2)	−0.07398 (17)	−0.3069 (2)	0.0655 (6)	
H5	0.4752	−0.0645	−0.3984	0.079*	
C6	0.56356 (18)	0.00679 (16)	−0.2258 (2)	0.0548 (5)	
H6	0.5489	0.0704	−0.2636	0.066*	
C7	0.67597 (17)	0.08183 (13)	−0.0024 (2)	0.0453 (5)	
H7	0.6967	0.0764	0.0986	0.054*	
C8	0.76952 (17)	0.32495 (13)	−0.04646 (19)	0.0415 (4)	
C9	0.81732 (17)	0.40832 (12)	0.04982 (19)	0.0404 (4)	
C10	0.86287 (18)	0.39728 (13)	0.1951 (2)	0.0455 (5)	
H10	0.8641	0.3347	0.2385	0.055*	
C11	0.90664 (18)	0.47857 (15)	0.2764 (2)	0.0534 (5)	
H11	0.9381	0.4694	0.3740	0.064*	
C12	0.90528 (18)	0.57268 (14)	0.2177 (2)	0.0527 (5)	
C13	0.8605 (2)	0.58265 (16)	0.0728 (3)	0.0707 (7)	
H13	0.8585	0.6454	0.0299	0.085*	
C14	0.8183 (2)	0.50216 (15)	−0.0111 (2)	0.0661 (6)	
H14	0.7903	0.5111	−0.1098	0.079*	
C15	0.9510 (2)	0.66110 (18)	0.3086 (3)	0.0776 (7)	
H15A	0.9073	0.6662	0.3930	0.116*	
H15B	0.9387	0.7205	0.2507	0.116*	
H15C	1.0362	0.6530	0.3402	0.116*	
H2	0.740 (2)	0.2368 (18)	0.1184 (11)	0.080*	

### Atomic displacement parameters (Å^2^)

**Table:**

	*U*^11^	*U*^22^	*U*^33^	*U*^12^	*U*^13^	*U*^23^
Cl1	0.1227 (6)	0.0485 (4)	0.0643 (4)	0.0043 (3)	−0.0136 (4)	0.0008 (3)
N1	0.0568 (10)	0.0352 (8)	0.0386 (9)	−0.0009 (7)	0.0025 (7)	−0.0062 (7)
N2	0.0665 (10)	0.0329 (8)	0.0319 (8)	−0.0025 (7)	0.0022 (8)	−0.0019 (7)
O1	0.0919 (11)	0.0446 (8)	0.0314 (7)	−0.0007 (7)	0.0065 (7)	0.0009 (6)
C1	0.0503 (11)	0.0411 (10)	0.0433 (11)	−0.0045 (8)	0.0118 (9)	−0.0052 (8)
C2	0.0595 (12)	0.0430 (11)	0.0510 (12)	−0.0032 (9)	0.0104 (10)	−0.0071 (9)
C3	0.0737 (14)	0.0397 (11)	0.0710 (15)	−0.0060 (10)	0.0132 (12)	−0.0104 (10)
C4	0.0755 (15)	0.0543 (14)	0.0714 (16)	−0.0171 (11)	0.0052 (13)	−0.0230 (12)
C5	0.0703 (15)	0.0673 (15)	0.0565 (13)	−0.0130 (11)	−0.0027 (11)	−0.0109 (11)
C6	0.0607 (12)	0.0499 (12)	0.0528 (12)	−0.0068 (10)	0.0028 (10)	−0.0039 (10)
C7	0.0591 (12)	0.0387 (10)	0.0380 (10)	−0.0013 (9)	0.0054 (9)	−0.0036 (8)
C8	0.0542 (11)	0.0357 (10)	0.0343 (10)	0.0069 (8)	0.0044 (8)	0.0016 (8)
C9	0.0508 (11)	0.0338 (9)	0.0373 (10)	0.0016 (8)	0.0074 (8)	0.0007 (7)
C10	0.0599 (12)	0.0358 (10)	0.0404 (11)	−0.0023 (8)	0.0033 (9)	0.0043 (8)
C11	0.0643 (13)	0.0512 (12)	0.0427 (11)	−0.0079 (10)	−0.0019 (9)	−0.0029 (9)
C12	0.0534 (12)	0.0444 (12)	0.0610 (13)	−0.0091 (9)	0.0099 (10)	−0.0072 (10)
C13	0.1038 (19)	0.0339 (11)	0.0721 (16)	−0.0111 (11)	0.0008 (14)	0.0086 (10)
C14	0.1066 (18)	0.0429 (12)	0.0456 (12)	−0.0066 (12)	−0.0057 (12)	0.0091 (10)
C15	0.0850 (17)	0.0563 (14)	0.0914 (19)	−0.0249 (12)	0.0092 (14)	−0.0196 (13)

### Geometric parameters (Å, º)

**Table:**

Cl1—C2	1.736 (2)	C7—H7	0.9300
N1—C7	1.269 (2)	C8—C9	1.486 (2)
N1—N2	1.379 (2)	C9—C10	1.376 (3)
N2—C8	1.353 (2)	C9—C14	1.380 (3)
N2—H2	0.895 (9)	C10—C11	1.379 (3)
O1—C8	1.227 (2)	C10—H10	0.9300
C1—C2	1.389 (3)	C11—C12	1.374 (3)
C1—C6	1.391 (3)	C11—H11	0.9300
C1—C7	1.462 (2)	C12—C13	1.370 (3)
C2—C3	1.382 (3)	C12—C15	1.505 (3)
C3—C4	1.368 (3)	C13—C14	1.377 (3)
C3—H3	0.9300	C13—H13	0.9300
C4—C5	1.371 (3)	C14—H14	0.9300
C4—H4	0.9300	C15—H15A	0.9600
C5—C6	1.374 (3)	C15—H15B	0.9600
C5—H5	0.9300	C15—H15C	0.9600
C6—H6	0.9300		
			
C7—N1—N2	116.71 (15)	O1—C8—C9	121.18 (16)
C8—N2—N1	117.93 (14)	N2—C8—C9	116.96 (15)
C8—N2—H2	121.8 (16)	C10—C9—C14	118.10 (17)
N1—N2—H2	119.9 (16)	C10—C9—C8	123.99 (15)
C2—C1—C6	116.78 (17)	C14—C9—C8	117.90 (16)
C2—C1—C7	122.13 (17)	C9—C10—C11	120.29 (17)
C6—C1—C7	121.09 (17)	C9—C10—H10	119.9
C3—C2—C1	121.96 (19)	C11—C10—H10	119.9
C3—C2—Cl1	117.91 (16)	C12—C11—C10	122.04 (18)
C1—C2—Cl1	120.10 (14)	C12—C11—H11	119.0
C4—C3—C2	119.5 (2)	C10—C11—H11	119.0
C4—C3—H3	120.2	C13—C12—C11	117.12 (18)
C2—C3—H3	120.2	C13—C12—C15	121.4 (2)
C3—C4—C5	119.95 (19)	C11—C12—C15	121.5 (2)
C3—C4—H4	120.0	C12—C13—C14	121.75 (19)
C5—C4—H4	120.0	C12—C13—H13	119.1
C4—C5—C6	120.4 (2)	C14—C13—H13	119.1
C4—C5—H5	119.8	C13—C14—C9	120.66 (19)
C6—C5—H5	119.8	C13—C14—H14	119.7
C5—C6—C1	121.4 (2)	C9—C14—H14	119.7
C5—C6—H6	119.3	C12—C15—H15A	109.5
C1—C6—H6	119.3	C12—C15—H15B	109.5
N1—C7—C1	119.45 (16)	H15A—C15—H15B	109.5
N1—C7—H7	120.3	C12—C15—H15C	109.5
C1—C7—H7	120.3	H15A—C15—H15C	109.5
O1—C8—N2	121.86 (16)	H15B—C15—H15C	109.5

### Hydrogen-bond geometry (Å, º)

**Table:**

*D*—H···*A*	*D*—H	H···*A*	*D*···*A*	*D*—H···*A*
N2—H2···O1^i^	0.90 (1)	2.05 (1)	2.8976 (19)	159 (2)

Symmetry code: (i) *x*, −*y*+1/2, *z*+1/2.
